# Abnormal rich club organization in end-stage renal disease patients before dialysis initiation and undergoing maintenance hemodialysis

**DOI:** 10.1186/s12882-020-02176-y

**Published:** 2020-11-26

**Authors:** Shaohui Ma, Ming Zhang, Yang Liu, Dun Ding, Peng Li, Xueying Ma, Hongjuan Liu, Junya Mu

**Affiliations:** 1 Department of Medical Imaging, First Affiliated Hospital of Xi’an Jiaotong University, No. 277, West Yanta Road, Xi’an, 710061 Shaanxi-Province People’s Republic of China; 2 Center for Brain Imaging, School of Life Science and Technology, Xidian University, Xi’an, 710126 People’s Republic of China; 3 Engineering Research Center of Molecular & Neuroimaging, Ministry of Education, Xi’an, 710126 People’s Republic of China; 4 Department of Medical Imaging, Second Affiliated Hospital of Xi’an Jiaotong University, Xi’an, 710061 People’s Republic of China; 5 Department of Medical Imaging, Shaanxi Nuclear Geology 215 Hospital, Xianyang, People’s Republic of China; 6 The Affiliated Hospital of Inner Mongolia Medical University, Hohhot, 010000 People’s Republic of China; 7 School of Life Science and Technology, Xidian University, Xi’an, 710071 People’s Republic of China

**Keywords:** End-stage renal disease, Dialysis initiation, Graph analyses, Cystatin C

## Abstract

**Background:**

End-stage renal disease (ESRD) patients are at a substantially higher risk for developing cognitive impairment compared with the healthy population. Dialysis is an essential way to maintain the life of ESRD patients. Based on previous research, there isn’t an uncontested result whether cognition was improved or worsened during dialysis.

**Methods:**

To explore the impact of dialysis treatment on cognitive performance, we recruited healthy controls (HCs), predialysis ESRD patients (predialysis group), and maintenance hemodialysis ESRD patients (HD group). All ESRD patients performed six blood biochemistry tests (hemoglobin, urea, cystatin C, Na+, K+, and parathyroid hormone). Neuropsychological tests were used to measure cognitive function. By using diffusion tensor imaging and graph-theory approaches, the topological organization of the whole-brain structural network was investigated. Generalized linear models (GLMs) were performed to investigate blood biochemistry predictors of the neuropsychological tests and the results of graph analyses in the HD group and predialysis group.

**Results:**

Neuropsychological analysis showed the HD group exhibited better cognitive function than the predialysis group, but both were worse than HCs. Whole-brain graph analyses revealed that increased global efficiency and normalized shortest path length remained in the predialysis group and HD group than the HCs. Besides, a lower normalized clustering coefficient was found in the predialysis group relative to the HCs and HD group. For the GLM analysis, only the Cystatin C level was significantly associated with the average fiber length of rich club connections in the predialysis group.

**Conclusions:**

Our study revealed that dialysis had a limited effect on cognitive improvement.

## Background

End-stage renal disease (ESRD) is known as the glomerular filtration rate of < 15 mL/min/1.73 m^2^, or continuous dialysis therapy is essential [1, 2]. Altered central nervous system function [3] and various neuropsychiatric troubles [4] were often found in ESRD patients, which are closely related to reducing the quality of life. One neuropsychological study documented that partial metabolites and excessive toxins could be removed when undergoing dialysis to retain effective basic life support [5]. Although some researchers pointed out that ESRD patients who are undergoing dialysis have universally been discovered to perform better than non-dialysis patients with ESRD on neuropsychological tests [6–12], others observed the persistence of cognitive dysfunction in ESRD patients on dialysis [6, 7, 9, 11–23]. Hence, it is necessary to explore the effect of dialysis on the pathophysiology of cognitive dysfunction in patients with ESRD.

Reproducible results of evidence observed cognitive decline in patients with chronic kidney disease and individuals during their dialysis therapy [5, 24–26]. Sabrina et al. discovered a reversible part of low neuropsychological testing in patients with ESRD who are receiving a single dialysis session, particularly in the abilities of memory, execution, and psychomotor speed [27]. However, regular dialysis treatment may abduct arterial hypoxemia, ephemeral hypotension, and undulations in electrolytes and brain water content, which may eventually result in an aberrant central nervous system [28]. Therefore, the effect of dialysis therapy in ESRD patients on cognitive dysfunction remains largely unclear.

Neuroimaging has proved to be a worthy tool to explore the neural mechanisms of whole brain cognitive impairments in continuous hemodialysis ESRD patients [29–31]. As we know, cognitive impairment does not only have the appearance of one or more altered isolated areas, but is also compactly associated with the abnormality of distributive brain circuits [32–34]. The human connectome is essential for research of basic neurobiology [35], which could help us to comprehensively and accurately describe the internal network connection pattern of the whole brain [36].

In our study, to explore the impact of dialysis treatment and kidney failure on cognitive performance, we recruited healthy controls (HCs), predialysis ESRD patients (predialysis group), and maintenance hemodialysis ESRD patients (HD group). Diffusion tensor imaging (DTI) was employed to systematically analyze the microstructure of the brain white matter network in all subjects. We hypothesized that both the predialysis group and HD group had aberrant topological organization of the brain anatomical network, and there was discrimination between the two groups. We further investigated the association among the resulting network abnormality, neuropsychological performance, and clinical blood tests of all participants.

## Methods

All prospective research was approved by the Medical Ethics Committee of the First Affiliated Hospital of the Medical College in Xi’an Jiao tong University and was conducted in accordance with the Declaration of Helsinki. Each participant signed a written informed consent before the experimental procedures were conducted.

### Participants

In our research, 27 HD patients (34.1 ± 1.66 years old), 32 age-, education- and gender-matched predialysis patients (32.7 ± 1.67 years old) and 33 HCs with normal sight (35.0 ± 1.72 years) underwent MR imaging. The dialysis duration was greater than 3 months in HD patients. The following exclusion criteria were employed: (1) macroscopic brain T2-visible lesions on MRI scans; (2) psychiatric disorders or major neurologic disorders; (3) ischemic diseases including acute ischemic cerebrovascular disease, acute peripheral arterial occlusion, and/or advanced liver or heart failure; (4) asymptomatic coronary ischemia by electrocardiogram testing; (5) a history of diabetes; (6) substance abuse including drugs, alcohol, and cigarettes; (7) color blindness; and (8) claustrophobia.

### Laboratory examinations

All patients with ESRD performed six blood biochemistry tests including hemoglobin, urea, cystatin C, Na^+^, K,^+^ and parathyroid hormone before MR imaging to evaluate renal function. No blood indicator was measured in HCs.

### Neuropsychological tests

All subjects underwent Auditory Verbal Learning Test–Huashan version (AVLT-H) to assess memory function, including immediate recall trial, short-term delayed recall trial, long-term delayed recall trial, and recognition trial. The neuropsychologic test contained 12 two-character spoken Chinese words from three diverse categories (occupations, apparel, flowers) with four words per category. The researcher read the list of words with 1 s between word intervals and asked each individual to remember as many words as possible [37]. AVLT-H scores were as follows: (1) immediate recall total score (IR-S), the total number of correct recollection of words in the first three learning tests; (2) short-term delayed recall score (SR-S), the total number of correct recollections of words when the researcher read the same word list 5 min after the first three learning tests; (3) long-term delayed recall score (LR-S), the total number of correct recollections of words when the researcher read the same word list 5 min after the first three learning tests; and (4) recognition score (REC-S), the total number of correct recollections of words when the researcher read the same word list and 12 unrelated words (occupations, apparel, flowers).

### Image acquisition

All MRI imaging data were acquired with a 3.0 Tesla GE Excite scanner using an eight-channel coil (GE Medical System, Milwaukee, WI), including a high-resolution T1-weighted image and diffusion tensor imaging (DTI) scans.

DTI sequences were obtained including 30 volume sequences with diffusion gradients applied along 30 non-collinear directions (b = 1000 s/mm^2^) and one volume sequence with a b value of 0 s/mm^2^, and the following parameters were slice thickness, 4 mm; field of view (FOV) = 240 × 240 mm^2^; matrix size = 128 × 128; repetition time (TR) = 40,000 ms; and echo time (TE) = 84 ms. Additionally, high-resolution anatomical images were also acquired by applying a T1-weighted three-dimensional MRI sequence with the following parameters: 140 axial slices; TR = 8.5 ms; TE = 3.4 ms; flip angle = 12°; slice thickness = 1.0 mm; no gap; matrix = 240 × 240; and FOV = 240 × 240 mm^2^. Each participant was placed in a standard head coil to decrease head movement during MRI data acquisition.

### Image preprocessing

Preprocessing of DTI imaging data included three steps. First, all raw DT imaging sequence quality was examined qualitatively. Then, the head motion and eddy current distortions were aligned by using an affine alignment of each diffusion-weighted image to the b = 0 image. Finally, brain extraction and diffusion tensor elements were evaluated by solving the Stejskal and Tanner equation [38, 39].

### Network construction

For each participant, a structural connectivity matrix was created by combining white matter tractography and cortical labels. In our study, we applied the Human Brainnetome Atlas [40] to extract the nodes of brain white matter connectivity, which segmented entire networks into 246 regions of interest. Additionally, the deterministic fiber tracking method [41] was employed to map white matter connections between brain regions. During the tracking procedure, we removed fibers which were less than 20 mm in length, as these may be false positive fibers [42]. For each participant, the two connectivity matrices described (1) unweighted networks (the number of fibers was greater than 3 between two brain regions), and (2) weighted networks along the average fibers strengths connecting a pair of ROIs.

### Network measures

The so-called rich club phenomenon of the brain network exists when the highly connected regions of a network are more intensively connected with each other than predicted on the basis of their high degree alone [43]. In recent studies, rich club organization of the brain structural connectome was discovered [30, 44–46]. In the current research, we employed the graph theory approach to analyze the rich club organization of the brain binary networks in all subjects at the range of the nodal degree (k) cutoff, including rich club coefficient (*φ*(*k*)), normalized *φ*(*k*) (*φ*_*norm*_(*k*)), clustering coefficient (*C*), shortest path length (*L*), global efficiency (*E*_*gob*_), and edge (*E*). The details and interpretations of these network measures are briefly described below.

The rich club coefficient, *φ*(*k*), which was defined as a ratio of the number of connections among nodal degree *k* or higher and the total probable number of connections if these regions were completely connected [44, 45].

This is defined as the fraction of edges, E, that connects nodes, N, of degree k or higher over a range of k-values:

$$ \varphi (k)=\frac{2{E}_{>k}}{N_{>k}\left({N}_{>k}-1\right)} $$

where k is the number of connections of node i, N > k is the number of nodal degree >*k* and E > k is the number of remaining connections that delete the regions with connections less than k. *φ*_*random*_(*k*) was calculated as the average on a set of 1000 random graphs within equal size and similar connectivity distribution. *φ*_*norm*_(*k*), which was computed as the fraction of *φ*(*k*) and *φ*_*random*_(*k*):

$$ {\varphi}_{norm}(k)=\frac{\varphi (k)}{\varphi_{random}(k)} $$

Therefore, *φ*_*random*_(*k*) could be summed up as a rich club organization usually being > 1.

To better evaluate the effect of the rich club organization, we added parameters at the same k-level range. By doing so, we could contrast and detect global parameters if the supporting standard metrics were altered across selected k-values or the entire k-value regime.

At a specific k value, *the clustering coefficient* of a node *i*, *C*_*i*_, which was defined as a ratio that is the proportion of possible connections that actually exist between the nearest neighbors of a node [47, 48]:

$$ {C}_i=\frac{2{e}_i}{k_i\left({k}_i-1\right)} $$

where *k*_*i*_ is the degree of node *i*, and *e*_*i*_ is the number actually existing between the nearest neighbors of node *i*.

The mean clustering coefficient of network C is the average of the clustering coefficient over all nodes, which indicates the extent of local interconnectivity or cliquishness in a network [47]:

$$ C=\frac{1}{N}{C}_i $$

*The shortest path length*, *L*_*i*, *j*_, defined as the minimal travel path for node *i* and node *j* in the network is computed as follows [47]:

$$ L=\frac{1}{N\left(N-1\right)}\sum \limits_{i\ne j}\mathit{\min}\left\{{L}_{i,j}\right\} $$

where N is the number of nodes in the network, and min {*L*_*i*, *j*_} is the shortest path length between any pair of nodes (e.g., node *i* and node *j*). The *L* of a network quantifies the ability for information propagation in parallel.

*The global efficiency* (*E*_*gob*_) of G that measures the capability of the parallel information transfer in the network [49] is defined as:

$$ {E}_{glob}(G)=\frac{1}{N\left(N-1\right)\;}\;\sum \limits_{i/j\in G}\frac{1}{L_{ij}} $$

where *L*_*i*, *j*_ is the shortest path length between node *i* and node *j* in G.

In order to normalize the parameters, we compared the actual values with the average calculated from 100 randomized networks of the same number of regions and nodal degree sequence. It can help to regulate these unstable graph theory indicators, such as C and L, as their absolute values provided a restricted message about the integration of the brain network [50]. Statistically, we performed the same analyses as described above for the rich club effect and its factors, N and E.

Additionally, we carried out the same analysis as described above for the number of the connections of the rich club organization across all k-levels, *E*.

### Network nodes and edges

In our study, we defined the rich club regions in three ways: on the basis of the group-average brain network which was calculated by the connections of all healthy subjects greater than 50%, on the individual level by ranking the degree value of the regions, and on the basis of the first 20% of most consistently interconnected regions in the HC groups [44].

Based on the definition of the rich club organization, we divided all nodes of the whole brain network into rich club and non-rich club nodes, and further divided the edges into three topological categories [45]: (1) the edges between two rich club regions were called rich club connections; (2) the edges between rich club regions and non-rich club regions were called feeder connections; and (3) the edges between two non-rich club regions were called local connections [45]. Additionally, for the brain weighted networks of the fiber length, we calculated the average value of the three types of connections for each participant.

### Statistical analysis

Software (SPSS version 16.0; SPSS, Chicago, Ill) was employed for the demographic and clinical characteristics among the HD group, predialysis group, and HC. To compare age, years of education, and neuropsychological tests, the one-way analysis of variance (one-way ANOVA) was applied among the three groups, and the independent-sample *t*-test was used between pairs of individual groups (HD group vs HCs, predialysis group vs HCs, and HD group vs predialysis group). The χ^2^ test was used to contrast gender distribution among the three groups. A significant difference was present if the *p*-value was less than 0.05.

To explore whether there were significant between-group differences in laboratory examinations (hemoglobin, urea, cystatin C, Na^+^, K,^+^ and parathyroid hormone) between the HD group and predialysis group, we employed the independent-sample *t*-test after controlling age, gender, and years of education. A *p* value of less than 0.05 was considered to show a significant difference.

For brain structural network connectivity (including *φ*(*k*), *φ*_*norm*_(*k*), clustering coefficient (*C*), shortest path length (*L*), global efficiency (*E*_*gob*_), and edge (*E*)), we employed a linear regression with coding controls as 0 and disease groups as 1 to test group differences for each group (HCs, HD group, and predialysis group) after removing age, gender, and education. The false discovery rate (FDR) approach was applied to correct for multiple comparisons across all k-level regimes. Additionally, we conducted the same method to analyze the differences of the average tract length of the rich club, feeder, and local connections among the three groups in the selected k level.

Generalized linear models (GLMs) were performed separately to investigate blood biochemistry predictors of the neuropsychological test and average tract length results in the two groups (HD group and predialysis group) with gender, age, and education imported as covariates. The threshold for a significant difference was set at an uncorrected *p* value of less than 0.05.

## Results

### Demographic, laboratory examinations and memory function comparisons

Demographic and laboratory examinations for the HD group, predialysis group, and HCs are summarized in Table 1. There was no significant difference in age (*p* = 0.532), education level (*p* = 0.541), and gender among the three groups (Table 1). Age, gender, and education were employed as covariates in a subsequent statistical analysis. Additionally, of all the results of blood indicator examinations between the two patient groups, hemoglobin, urea, Na,^+^ and parathyroid hormone were thought to show significant differences (*p* < 0.05), but no difference was indicated for Cystatin C and K^+^ (*p* > 0.05) (Table 1).

**Table 1:** Demographic and clinical characteristics of healthy controls and patients with end-stage renal disease

Variable	mean ± SE			F-value			
	HCs	predialysis group	HD group		P1	P2	P3
**Demographic**							
N	33	32	27	–	–	–	–
Age (years)	35.0 ± 1.72	32.7 ± 1.67	34.1 ± 1.66	0.532	0.715	0.322	0.512
Gender (M/F)	15/18	17/15	14/13	–	0.796	0.901	0.696
Education (years)	12.3 ± 0.50	12.0 ± 1.01	11.2 ± 0.55	0.541	0.15	0.787	0.523
Dialysis duration	–	–	30 ± 3.22	***–***	–	–	–
**Laboratory Examinations**							
Hemoglobin(g/L)	**–**	86.3 ± 3.51	103.7 ± 3.87	***–***	–	–	0.0016*
Urea (μmol/L)	**–**	31.4 ± 1.83	23.5 ± 1.57	***–***	–	–	0.0022*
Cystatin C (mg/L)	**–**	4.4 ± 0.616	4.4 ± 0.68	–	–	–	0.989
Na + (mmol/L)	**–**	139.8 ± 0.57	144.0 ± 0.68	***–***	–	–	< 0.001*
K+ (mmol/L)	**–**	4.6 ± 0.12	5.0 ± 0.13	–	–	–	0.0673
Parathyroid hormone (pg/mL)	**–**	303.3 ± 30.37	731.0 ± 88.78	***–***	***–***	***–***	< 0.001*

P1:HCs vs. HD group; P2:HCs vs. predialysis group; P3:HD group vs. predialysis group. The two group differences in subjects’ basic information were analyzed using a two-sample t-test. The numbers of males and females were analyzed using a χ2 test. *p < 0.05

*ESRD* end-stage renal disease, *HCs* healthy controls, *predialysis group* predialysis ESRD patients, *HD group* maintenance hemodialysis ESRD patients

Higher IR-S and SR-S were found in HCs compared with ESRD patients, and the HD group compared with the predialysis group (Table 2, all *p* < 0.05). LR-S and REC-S performed better in HCs compared with ESRD patients (*p* < 0.05), while no difference was discovered between the HD group and predialysis group (*p* > 0.05) (Table 2).

**Table 2:** Neuropsychologic tests of healthy controls and patients with end-stage renal disease

Variable	mean ± SE			F-value	***P***1-value	***P***2-value	***P***3-value
	HCs	HD group	predialysis group				
IR-S	26.8 ± 0.79	24.0 ± 0.87	20.0 ± 0.92	16.708*	0.017*	< 0.001*	0.003*
SR-S	10.3 ± 0.24	9.2 ± 0.35	7.9 ± 0.45	12.300*	0.010*	< 0.001*	0.028*
LR-S	10.3 ± 0.28	8.15 ± 0.34	7.8 ± 0.49	13.092*	< 0.001*	< 0.001*	0.521
REC-S	11.8 ± 0.10	11.2 ± 0.19	11.1 ± 0.27	3.286*	0.006*	0.029*	0.860

P1:HCs vs. HD group; P2:HCs vs. predialysis group; P3:HD group vs. predialysis group. The two group differences in subjects’ neuropsychologic tests were analyzed using a two-sample t-test. *p < 0.05

*IR-S* immediate recall total score, *SR-S* short-term delayed recall score, *LR-S* long-term delayed recall score, *REC-S* recognition score, *ESRD* end-stage renal disease, *HCs* healthy controls, *predialysis group* predialysis ESRD patients, *HD group* maintenance hemodialysis ESRD patients

After the GLM analysis, we discovered a significant correlation between Cystatin C and IR-S, SR-S and LR-S in the predialysis group (uncorrected *p* < 0.05) (Table 3).

**Table 3:** Multivariable generalized linear models for prediction of neuropsychological tests

Variable	IR-S			SR-R			LR-S			REC-S		
	SE	wald	*P*	SE	wald	*P*	SE	wald	*P*	SE	wald	*P*
**HD group**												
Hemogloblin(g/L)	0.043	0.519	0.471	0.017	5.127	0.024*	0.017	3.770	0.052	0.009	0.102	0.750
Urea (μmol/L)	0.107	2.990	0.084	0.041	4.598	0.032*	0.043	2.128	0.145	0.022	3.628	0.057
Cystatin (mg/L)	0.722	1.228	0.268	0.279	0.754	0.385	0.291	0.000	0.989	0.149	0.379	0.538
Na + (mmol/L)	0.231	0.325	0.569	0.089	0.134	0.715	0.093	0.561	0.454	0.048	0.015	0.902
K+ (mmol/L)	1.207	1.890	0.169	0.466	0.744	0.389	0.487	0.001	0.972	0.250	4.394	0.036*
parathyroid hormone (pg/mL)	0.002	0.362	0.547	0.001	0.530	0.467	0.001	0.416	0.519	0.000	1.046	0.306
**predialysis group**												
Hemogloblin(g/L)	0.059	0.055	0.814	0.028	0.175	0.675	0.028	0.004	0.953	0.018	0.271	0.603
Urea (μmol/L)	0.102	1.394	0.238	0.048	1.379	0.240	0.049	3.628	0.057	0.031	2.207	0.155
Cystatin C (mg/L)	1.213	4.045	0.044*	0.574	4.614	0.032*	0.580	6.199	0.013*	0.364	2.677	0.102
Na + (mmol/L)	0.355	0.551	0.458	0.168	1.317	0.251	0.170	2.640	0.104	0.107	1.070	0.301
K+ (mmol/L)	1.334	1.504	0.220	0.631	4.209	0.040*	0.638	2.970	0.085	0.400	0.910	0.340
parathyroid hormone (pg/mL)	0.006	0.114	0.736	0.003	0.739	0.390	0.003	0.996	0.318	0.002	2.023	0.155

*IR-S* immediate recall total score, *SR-S* short-term delayed recall score, *LR-S* long-term delayed recall score, *REC-S* recognition score, *ESRD* end-stage renal disease, *HCs* healthy controls, *predialysis group* predialysis ESRD patients, *HD group* maintenance hemodialysis ESRD patients

* uncorrected *p* value of less than 0.05

### Global topologic organization of the structural network

**Fig. 1:**
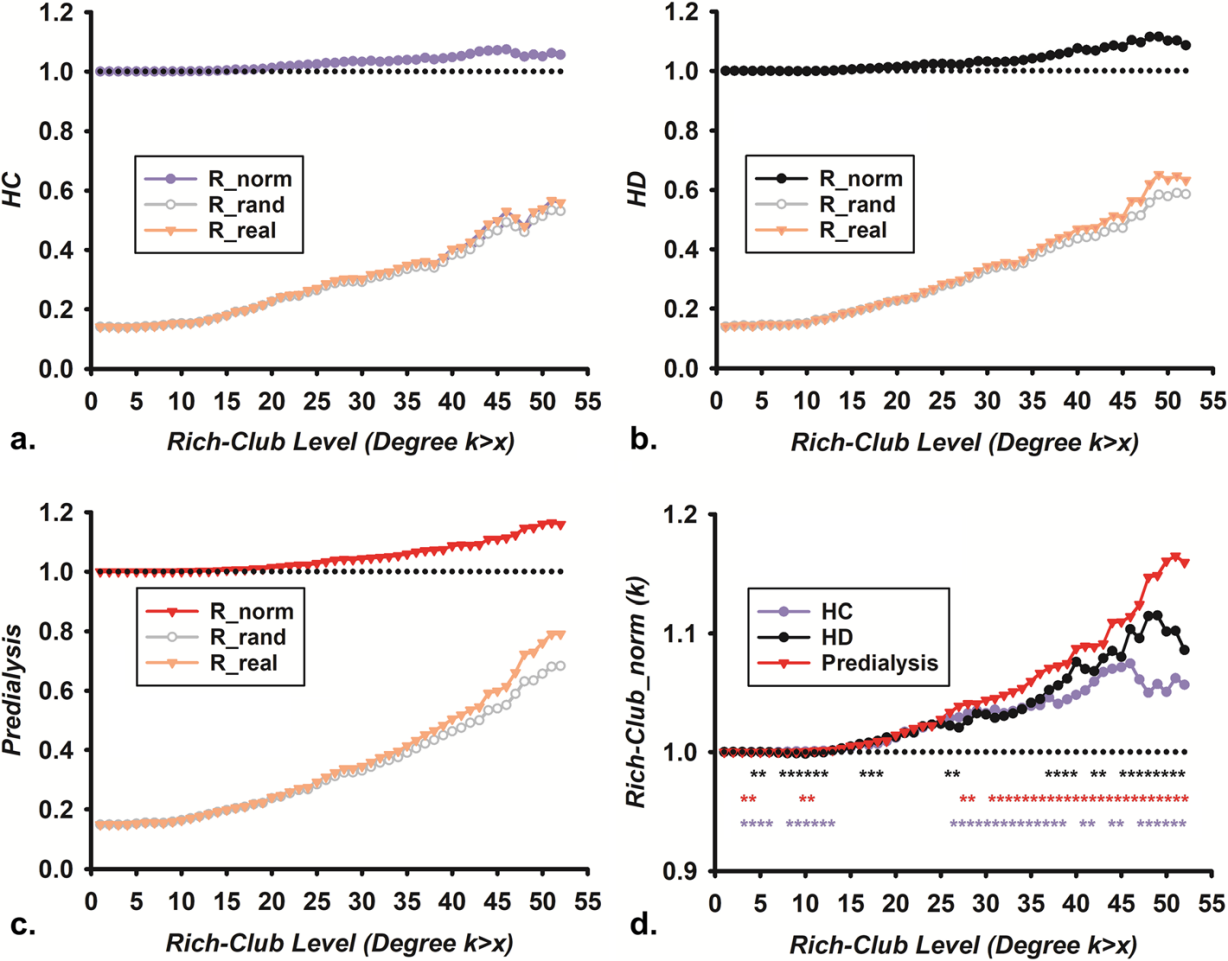
The rich club phenomenon in HCs, HD group and predialysis group. **a**. the rich club level of HCs. **b**. the rich club level of the HD group. **c**. the rich club level of the predialysis group. **d** The rich club phenomenon in HCs, HD group, and predialysis group. ESRD: End-stage renal disease; predialysis group:predialysis ESRD patients; HD group: ESRD patients undergoing maintenance hemodialysis; HCs:healthy controls

**Fig. 2:**
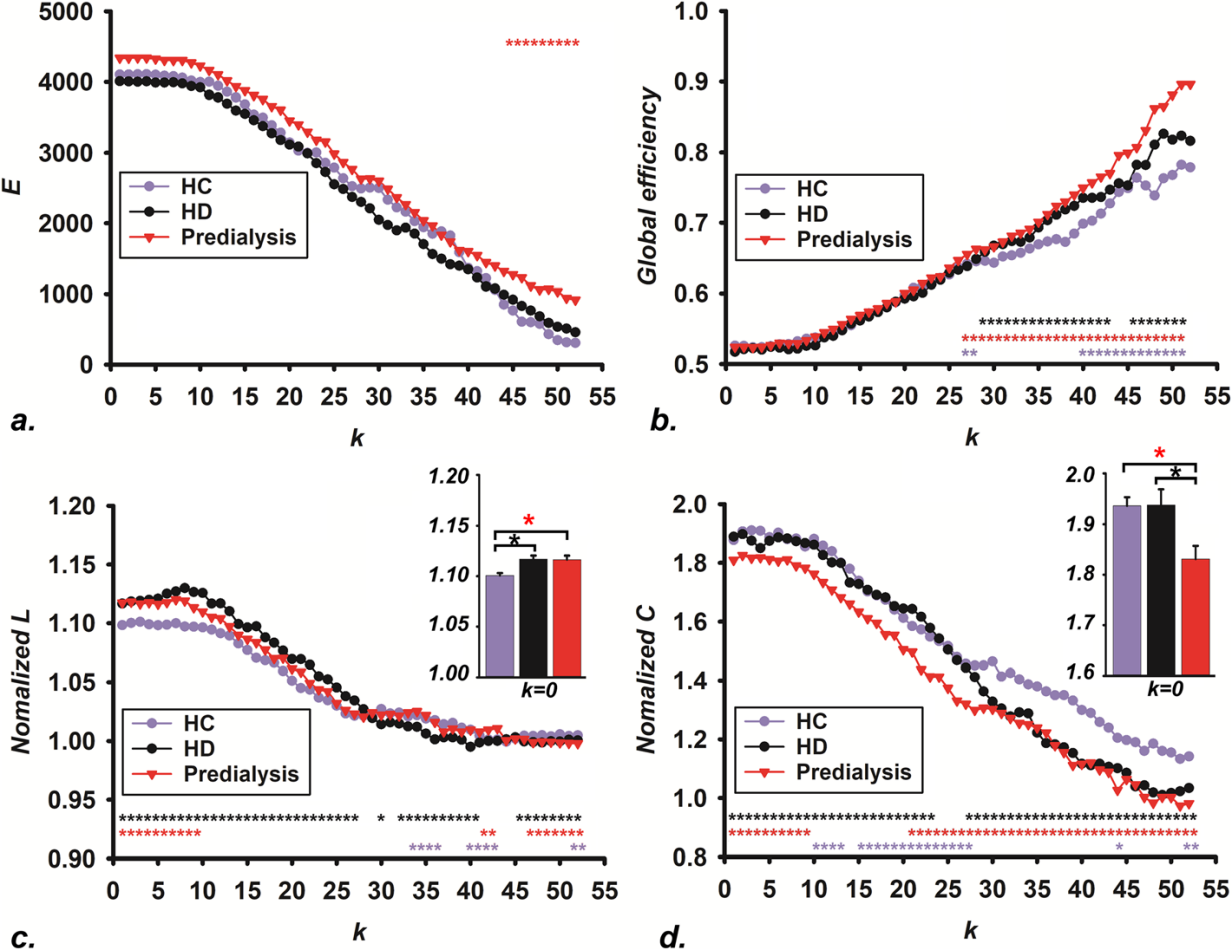
The difference of network properties among HCs, HD group, and predialysis group. **a**. the E values of three groups at different k levels. **b** the global efficiency of the three groups at different k levels. **c** the normalized L of three groups at different k levels. **d** the normalized C of the three groups at different k levels

In the global brain network, φ_norm_(k) was increasingly higher than 1 over an increasing regime of k-levels among the three groups (HCs, HD group, and predialysis group), which exhibited that a rich club phenomenon was detectable. The φ_norm_(k) was significantly greater in patients with ESRD than in the HC group, and the predialysis group was higher than the HD group for φ_norm_(k) at a steady range of k-values (*p* < 0.05, FDR corrected, Fig. 1). *E*, global efficiency, normalized shortest path length (normalized L), and normalized clustering coefficients (normalized C) were considered to show significant differences for each group (HCs, HD group, and predialysis group) across high-degree k-levels respectively (*p* < 0.05, FDR corrected, Fig. 2). Additionally, compared with HCs, both the HD group and predialysis group exhibited significantly increased normalized L at k = 0, with no significant differences between the HD group and predialysis group (*p* < 0.05, FDR corrected, Fig. 2c). There were evidently lower normalized C in the predialysis group relative to the HCs and HD group, but no significant difference was discovered for normalized C between the HCs and HD group individuals at k = 0 (*p* < 0.05, FDR corrected, Fig. 2d). Here, graph analysis research recorded the differences of the rich club organization for the brain structural network.

### Regional topologic organization of the structural network

The results of rich club regions were convergent on a group-level, on the individual level, and across the group of participants, including the insular gyrus (INS), cingulate gyrus (CG), superior frontal gyrus (SFG), middle frontal gyrus (MFG), superior parietal lobule (SPL), inferior parietal lobule (IPL), superior temporal gyrus (STG), middle temporal gyrus (MTG), precentral gyrus (PrG), precuneus (Pcun), caudal cuneus gyrus (Cun), occipital polar cortex (OcG), superior occipital gyrus (sOcG), hippocampus (Hipp), basal ganglia (Str), and thalamus (Tha).

**Fig. 3:**
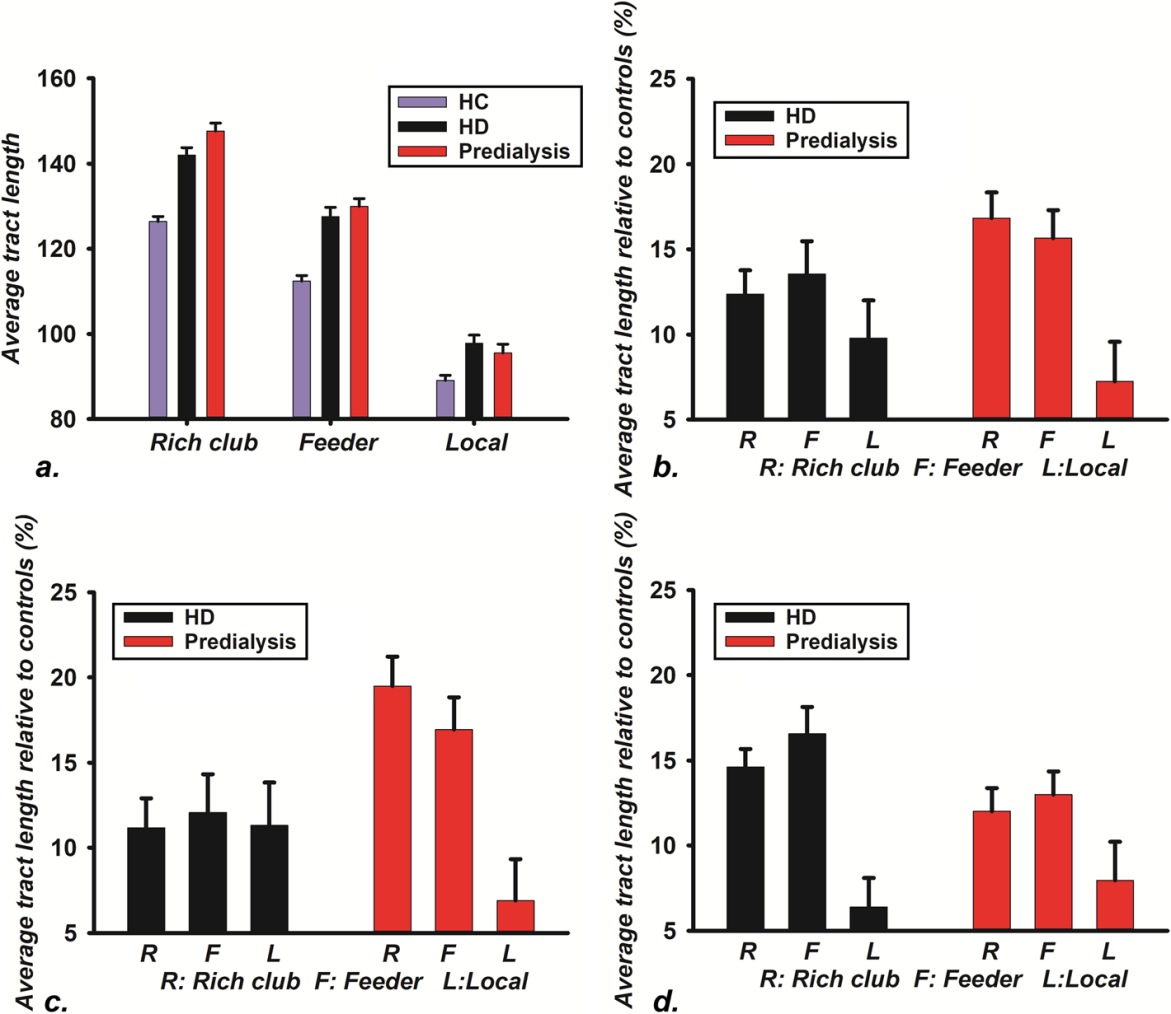
The difference of edges of the rich club among HCs, HD group, and predialysis group

In our results, we observed significantly increased average fiber length of the rich club, feeder, and local connections in both the HD group and predialysis group relative to HCs (*p* < 0.05, FDR corrected, Fig. 3). The average fiber length of the rich club connections of the predialysis group showed a significant increase relative to the HD group, with no differences for the average tract length of the feeder and local connections (*p* < 0.05, FDR corrected, Fig. 3).

### Relationships between average fiber length and laboratory examinations

GLM analysis revealed that the Cystatin C level was significantly associated with the average fiber length of the rich club connections in the predialysis group (uncorrected *p* < 0.05), with no relationship for the average tract length of the feeder and local connections (*p* > 0.05) (Table 4). There was no correlation between the Cystatin C level and the average fiber length of the three types of brain structural connections in the HD group (*p* > 0.05) (Table 4).

**Table 4:** Multivariable generalized linear models for prediction of average fiber length

Variable	Rich club			Feeder			Local		
	SE	wald	*P*	SE	wald	*P*	SE	wald	*P*
**HD group**									
Hemogloblin(g/L)	0.094	3.058	0.080	0.114	4.373	0.037*	0.104	3.317	0.069
Urea (μmol/L)	0.233	0.048	0.827	0.281	0.019	0.889	0.258	0.156	0.693
Cystatin C (mg/L)	1.577	0.479	0.489	1.898	0.736	0.391	1.743	0.999	0.318
Na + (mmol/L)	0.505	0.618	0.432	0.608	0.602	0.438	0.558	0.000	0.985
K+ (mmol/L)	2.638	0.009	0.925	3.175	0.000	1.000	2.914	0.002	0.961
parathyroid hormone (pg/mL)	0.004	0.644	0.422	0.005	0.760	0.383	0.004	0.536	0.464
**predialysis group**									
Hemogloblin(g/L)	0.109	0.367	0.545	0.113	1.414	0.234	0.140	0.594	0.441
Urea (μmol/L)	0.190	2.251	0.134	0.196	1.038	0.308	0.244	0.135	0.713
Cystatin C (mg/L)	2.256	6.601	0.010*	2.333	1.924	0.165	2.898	0.182	0.670
Na + (mmol/L)	0.660	0.209	0.648	0.683	0.824	0.364	0.848	2.960	0.085
K+ (mmol/L)	2.482	6.313	0.012*	2.567	3.457	0.063	3.188	0.393	0.531
parathyroid hormone (pg/mL)	0.011	4.035	0.045*	0.011	0.920	0.337	0.014	0.063	0.802

*predialysis group* predialysis ESRD patients, *HD group* maintenance hemodialysis ESRD patients, *ESRD* end-stage renal disease

* an uncorrected *p* value of less than 0.05

## Discussion

In our study, we detected the relationship between dialysis treatment, kidney failure, and cognitive performance in ESRD patients. This study demonstrated that dialysis treatment has a limited protection effect on both cognitive performance and brain networks in ESRD patients, which can be summarized as follows: (i) The IR-S and SR-S comply with the order: HCs > HD group>predialysis group; the LR-S and REC-S comply with the order: HCs > HD group = predialysis group; (ii) For the global topologic organization of the structural network, both the HD group and predialysis group have reduced the information transfer efficiency compared to HCs; and (iii) Cystatin C level was found to be correlated with the average fiber length of rich club connections in the predialysis group.

We found that the HD group exhibited better memory than the predialysis group, but both were worse than HCs. Based on previous research, they were not able to provide an uncontested result whether cognition was improved or worsened during dialysis [51–53]. Based on the results of the neuropsychological tests, our results seemed to indicate that ESRD patients who underwent long-term dialysis have better cognitive function than ESRD patients before dialysis initiation. Dialysis had an improvement effect for cognitive function. However, there are also many studies which found worse cognitive function over time for dialysis patients [6, 21]. The reason may be concerned with the time of dialysis. Because some reports have indicated that the duration of dialysis was also an important feature for cognition [21]. The mean duration of dialysis for our subjects was 30 months, with the beneficial effect of treatment on retaining cognition lasting only a limited amount of time. At some point in a longer duration, the gradient course of cognition might reach a plateau or even decline, but we cannot account for this in this study.

By using graphic metrics, such as efficiency and modularity, we found that all of the groups (HCs, HD group, and predialysis group) exhibited a rich club phenomenon. Increased global efficiency remained in the predialysis group and HD group rather than the HCs. Global efficiency measures the ability of parallel information propagation within a network. Higher global efficiency seems to indicate effective integrity and rapid information propagation between and across remote brain regions [54, 55]. For our earlier studies, decreased global efficiency has previously been reported in functional brain networks [56]. Here, increased global efficiency in the structural network may suggest self-regulation of the brain. A long tradition of research has clearly shown the brain’s ability to learn volitional control of its own activity and effects on behavior [57]. The increased global efficiency in structural networks and decreased global efficiency in functional network synergy were used to maintain brain functioning. Besides, both the HD group and predialysis group exhibited significantly increased normalized L at k = 0 than healthy control subjects. It indicated that, to maintain brain function, the structural network of ESRD patients (no matter if undergoing dialysis) may show a network recombination to connect more distant brain regions. Dialysis seems not to have a protective effect on the brain network. Furthermore, we found lower normalized C in the predialysis group relative to the HCs and HD group, but no significant difference was discovered between the HCs and HD group. It suggested that patients undergoing long-term dialysis have better cognitive function than ESRD patients before dialysis initiation. From the above information, our findings have implications that dialysis has a limited protective effect for the brain network.

For the correlation analysis between the structural network and laboratory examinations, only the Cystatin C level was significantly associated with the average fiber length of rich club connections in the predialysis group. Many studies have reported that Cystatin C is one of the early markers of chronic kidney disease which might serve as early and effective markers for cognitive decline in kidney patients [58, 59]. Besides, studies have shown that the Cystatin C concentration is also associated with the risk of dementia [60, 61]. For the neuropsychological tests, the difference between the HD group and predialysis group mainly focuses on memory. Higher levels of Cystatin C may play a role in worse memory scores, which is consistent with our results and previous studies [60, 61].

Our study had several limitations which need to be addressed in future studies. First, the sample size is relatively small in this study, which limits efforts in the statistical analysis. A large group of population samples is needed in the future to verify the relationships between dialysis treatment and cognitive performance. Second, blood biochemistry levels were only tested in ESRD patients, but blood biochemistry tests should be considered in healthy control subjects to investigate the relationship between the network and blood biochemistry in further studies. Besides, we only selected six blood biochemistry values in our study, and a more detailed biochemistry test should preformed in a further study. Third, the effect of anemia on cognitive was not assessed in our study. Finally, the neuropsychological tests in our study were all based on a scale test, and more comprehensive cognitive-ability tasks should be obtained in further experiments.

## Conclusion

To summarize, we conclude that cognitive function seems to improve in ESRD patients who underwent dialysis treatment. Dialysis treatment may predict better cognitive performance, but the improvement effect is limited. Cystatin C level has a potential relationship between cognitive function and brain function in ESRD patients before dialysis initiation. These results highlighted the need for a better understanding of dialysis treatment on cognition. In the future, the relationship between cognitive function and the different stage and duration of dialysis should be observed.

## Data Availability

The datasets used and/or analyzed during the current study are available from the corresponding author upon reasonable request.

## Abbreviations

ESRD: End-stage renal disease
HD: Hemodialysis
predialysis group: Predialysis ESRD patients
HD group: ESRD patients undergoing maintenance hemodialysis
HCs: Healthy controls
IR-S: Immediate recall total score
SR-S: Short-term delayed recall score
LR-S: Long-term delayed recall score
REC-S: Recognition score

## References

[1] Luo S, Qi RF, Wen JQ, Zhong JH, Kong X, Liang X, Xu Q, Zheng G, Zhang Z, Zhang LJ (2016). Abnormal intrinsic brain activity patterns in patients with end-stage renal disease undergoing peritoneal dialysis: a resting-state functional MR imaging study. Radiology.

[2] Kim HS, Park JW, Bai DS, Jeong JY, Hong JH, Son SM, Jang SH (2011). Diffusion tensor imaging findings in neurologically asymptomatic patients with end stage renal disease. NeuroRehabilitation.

[3] Deyn PPD, Saxena VK, Abts H, Borggreve F, Crols R (1992). Clinical and pathophysiological aspects of neurological complications in renal failure. Acta Neurol Belg.

[4] Brouns R, Deyn PPD (2004). Neurological complications in renal failure: a review. Clin Neurol Neurosurg.

[5] Williams MA, Sklar AH, Burright RG, Donovick PJ (2004). Temporal effects of dialysis on cognitive functioning in patients with ESRD. Am J Kidney Dis.

[6] Bo H (1974). A prospective study of patients in chronic hemodialysis—III. Predictive value of intelligence, cognitive deficit and ego defence structures in rehabilitation. J Psychosom Res.

[7] Ginn HE, Teschan PE, Walker PJ, Bourne JR, Hamel B (1975). Neurotoxicity in uremia. Kidney Int Suppl.

[8] Murawski BJ (1975). Psychological approaches to study the uremic state. Kidney Int Suppl.

[9] Teschan PE, Ginn HE, Bourne JR, Ward JW, Hamel B, Nunnally JC, Musso M, Vaughn WK (1979). Quantitative indices of clinical uremia. Kidney Int.

[10] Ryan JJ, Souheaver GT, Dewolfe AS (1981). Halstead-reitan test results in chronic hemodialysis. J Nerv Ment Dis.

[11] Mckee DC, Burnett GB, Raft DD, Batten PG, Bain KP (1982). Longitudinal study of neuropsychological functioning in patients on chronic hemodialysis: a preliminary report. J Psychosom Res.

[12] Baker LRI, Brown AL, Byrne J, Charlesworth M, Warrington EK (1989). Head scan appearances and cognitive function in renal failure. Clin Nephrol.

[13] Greenberg RP, Davis G, Massey R (1973). The psychological evaluation of patients for a kidney transplant and hemodialysis program. Am J Psychiatry.

[14] Teschan PE, Ginn HE, Walker PJ, Bourne JR, Ward JW (1974). Quantified functions of the nervous system in uremic patients on maintenance dialysis. Trans Am Soc Artif Intern Organs.

[15] Spehr W, Sartorius H, Berglund K, Hjorth B, Kablitz C, Plog U, Wiedemann PH, Zapf K (1977). EEG and haemodialysis. A structural survey of EEG spectral analysis, Hjorth's EEG descriptors, blood variables and psychological data. Electroencephalogr Clin Neurophysiol.

[16] English A, Savage RD, Britton PG, Ward MK, Kerr DN (1978). Intellectual impairment in chronic renal failure. Br Med J.

[17] Alexander L, Hightower MG, Anderson RP, Snow NE (1980). Suitablity of vigilance test data as a neurobehavioral measure of uremic status. Percept Mot Skills.

[18] Harold AZ, Patrick EL, Sarah MMC (1980). Psychological measurement of memory deficits in dialysis patients. Percept Mot Skills.

[19] Gilli P, Bastiani P (1983). De: cognitive function and regular dialysis treatment. Clin Nephrol.

[20] Ratner DP, Adams KM, Levin NW, Rourke BP (1983). Effects of hemodialysis on the cognitive and sensory-motor functioning of the adult chronic hemodialysis patient. J Behav Med.

[21] Wolcott DL, Wellisch DK, Marsh JT, Schaeffer J, Landsverk J, Nissenson AR (1988). Relationship of dialysis modality and other factors to cognitive function in chronic dialysis patients. Am J Kidney Dis.

[22] Marsh JT, Brown WS, Wolcott D, Carr CR, Harper R, Schweitzer SV, Nissenson AR (1991). rHuEPO treatment improves brain and cognitive function of anemic dialysis patients. Kidney Int.

[23] Churchill DN, Bird DR, Taylor W, Beecroft ML, Gorman J, Wallace JE (1992). Effect of high-flux hemodialysis on quality of life and neuropsychological function in chronic hemodialysis patients. Am J Nephrol.

[24] Kurella M (2005). Chronic kidney disease and cognitive impairment in the ederly: the health, aging, and body composition study. J Am Soc Nephrol Jasn.

[25] Chilcot J, Wellsted D, Silva-Gane MD, Farrington K (2008). Depression on dialysis. Nephron Clin Pract.

[26] Ma X, Jiang G, Li S, Wang J, Zhan W, Zeng S, Tian J, Xu Y (2015). Aberrant functional connectome in neurologically asymptomatic patients with end-stage renal disease. PLoS One.

[27] Schneider SM, Malecki AK, Katrin M, Robby S, Matthias G, Peter M, Marcus H, Heike K, Kristin J, Kielstein JT (2015). Effect of a single dialysis session on cognitive function in CKD5D patients: a prospective clinical study. Nephrol Dial Transplant.

[28] Toyoda (2002). Simultaneous onset of haemorrhagic and ischaemic strokes in a haemodialysis patient. J Neurol Neurosurg Psychiatry.

[29] Prohovnik I, Post J, Uribarri J, Lee H, Sandu O, Langhoff E (2007). Cerebrovascular effects of hemodialysis in chronic kidney disease. J Cereb Blood Flow Metab.

[30] Qiu Y, Lv X, Su H, Jiang G, Cheng L, Tian J, Satoru H (2014). Structural and functional brain alterations in end stage renal disease patients on routine hemodialysis: a voxel-based morphometry and resting state functional connectivity study. PLoS One.

[31] Zhang R, Liu K, Yang L, Zhou T, Qian S, Li B, Peng Z, Li M, Sang S, Jiang Q (2015). Reduced white matter integrity and cognitive deficits in maintenance hemodialysis ESRD patients: a diffusion-tensor study. Eur Radiol.

[32] Liu J, Liang J, Qin W, Tian J, Yuan K, Bai L, Zhang Y, Wang W, Wang Y, Li Q (2009). Dysfunctional connectivity patterns in chronic heroin users: an fMRI study. Neurosci Lett.

[33] Ling-Li Z, Hui S, Li L, Wang L, Li B, Peng F, Zhou Z, Li Y, Hu D (2012). Identifying major depression using whole-brain functional connectivity: a multivariate pattern analysis. Brain A J Neurol.

[34] Nan J, Liu J, Li G, Xiong S, Yan X, Yin Q, Zeng F, Deneen KMV, Liang F, Gong Q (2013). Whole-brain functional connectivity identification of functional dyspepsia. PLoS One.

[35] Toga AW, Clark KA, Thompson PM, Shattuck DW, VHJ D (2012). Mapping the human connectome. Neurosurgery.

[36] Varela FJ, Lachaux JP, Rodriguez E, Martinerie JF (2001). The brainweb: phase synchronization and large-scale integration. Nat Rev Neurosci.

[37] Zhao Q, Lv Y, Yan Z, Zhen H, Guo Q, Sonia B (2012). Short-term delayed recall of auditory verbal learning test is equivalent to long-term delayed recall for identifying amnestic mild cognitive impairment. PLoS One.

[38] Basser PJ, Mattiello J, LeBihan D (1994). MR diffusion tensor spectroscopy and imaging. Biophys J.

[39] Basser PJ, Pierpaoli C (1996). Microstructural and physiological features of tissues elucidated by quantitative-diffusion-tensor MRI. J Magn Reson.

[40] Fan L, Li H, Zhuo J, Zhang Y, Wang J, Chen L, Yang Z, Chu C, Xie S, Laird AR (2016). The human brainnetome atlas: a new brain atlas based on connectional architecture. Cereb Cortex.

[41] Yeh F-C, Verstynen TD, Wang Y, Fernández-Miranda JC (2013). Deterministic diffusion fiber tracking improved by quantitative anisotropy. PLoS One.

[42] Daianu M, Mezher A, Mendez MF, Jahanshad N, Jimenez EE, Thompson PM (2016). Disrupted rich club network in behavioral variant frontotemporal dementia and early-onset Alzheimer's disease. Hum Brain Mapp.

[43] Colizza V, Flammini A, Serrano MA, Vespignani A (2006). Detecting rich-club ordering in complex networks. Nat Phys.

[44] van den Heuvel MP, Sporns O (2011). Rich-club organization of the human connectome. J Neurosci.

[45] van den Heuvel MP, Kahn RS, Goni J, Sporns O (2012). High-cost, high-capacity backbone for global brain communication. Proc Natl Acad Sci U S A.

[46] van den Heuvel MP, Sporns O (2013). An anatomical substrate for integration among functional networks in human cortex. J Neurosci.

[47] Watts DJ, Strogatz SH (1998). Collective dynamics of small world networks. Nature.

[48] Liu J, Zhao L, Li G, Xiong S, Nan J, Li J, Yuan K, von Deneen KM, Liang F, Qin W (2012). Hierarchical alteration of brain structural and functional networks in female migraine sufferers. PLoS One.

[49] Latora V, Marchiori M (2001). Efficient behavior of small-world networks. Phys Rev Lett.

[50] Lock T (2012). Networks of the brain. J Dev Behav Pediatr.

[51] Investigators CS (2018). Cognitive impairment in non–dialysis-dependent CKD and the transition to dialysis: findings from the chronic renal insufficiency cohort (CRIC) study. Am J Kidney Dis.

[52] Song M-K, Paul S, Ward SE, Gilet CA, Hladik GA (2018). One-year linear trajectories of symptoms, physical functioning, cognitive functioning, emotional well-being, and spiritual well-being among patients receiving dialysis. Am J Kidney Dis.

[53] Neumann D, Mau W, Wienke A, Girndt M (2017). Peritoneal dialysis is associated with better cognitive function than hemodialysis over a one-year course. Kidney Int.

[54] Sporns O, Zwi JD (2004). The small world of the cerebral cortex. Neuroinformatics.

[55] Wang J, Wang X, He Y, Yu X, Wang H, He Y (2015). Apolipoprotein E epsilon 4 modulates functional brain connectome in alzheimer's disease. Hum Brain Mapp.

[56] Mu J, Chen T, Liu Q, Ding D, Ma X, Li P, Li A, Huang M, Zhang Z, Liu J (2017). Abnormal interaction between cognitive control network and affective network in patients with end-stage renal disease. Brain Imaging Behav.

[57] Birbaumer N, Ruiz S, Sitaram R (2013). Learned regulation of brain metabolism. Trends Cogn Sci.

[58] Wei Y, Wei YK, Zhu J (2017). Early markers of kidney dysfunction and cognitive impairment among older adults. J Neurol Sci.

[59] Yaffe K, Kurella-Tamura M, Ackerson L, Hoang TD, Anderson AH, Duckworth M, Go AS, Krousel-Wood M, Kusek JW, Lash JP (2014). Higher levels of cystatin C are associated with worse cognitive function in older adults with chronic kidney disease: the chronic renal insufficiency cohort cognitive study. J Am Geriatr Soc.

[60] Yelena S, Peters KW, Areef I, Kristine Y, Fink HA, Stone KL, Michael S, Ensrud KE (2014). Fractures ftSoO: cystatin C and cognitive impairment 10 years later in older women. J Gerontol A Biol Sci Med Sci.

[61] Yaffe K, Lindquist K, Shlipak MG, Simonsick E, Kurella-Tamura M (2008). Cystatin-C as a marker of cognitive function in elders: findings from the health ABC study. Ann Neurol.

